# Endobronchial ultrasound-guided transbronchial needle aspiration (EBUS-TBNA) in diagnosis of mediastinal lesions

**DOI:** 10.1590/S1679-45082018AO4094

**Published:** 2018-05-03

**Authors:** Ricardo Sales dos Santos, Marcia Jacomelli, Juliana Pereira Franceschini, Iunis Suzuki, Altair da Silva Costa, Christina Shiang, Addy Lidvina Mejia Palomino

**Affiliations:** 1 Hospital Israelita Albert Einstein, São Paulo, SP, Brazil; 2 Hospital das Clínicas, Faculdade de Medicina, Universidade de São Paulo, São Paulo, SP, Brazil; 3 Centro Universitário São Camilo, São Paulo, SP, Brazil; 4 Universidade Federal de São Paulo, São Paulo, SP, Brazil

**Keywords:** Mediastinum, Biopsy, fine-needle, Endoscopic ultrasound-guided fine needle aspiration/methods, Ultrasonography, interventional, Lymph nodes, Bronchoscopy, Mediastino, Biópsia por agulha fina, Aspiração por agulha fina guiada por ultrassom endoscópico/métodos, Ultrassonografia de intervenção, Linfonodos, Broncoscopia

## Abstract

**Objective:**

To describe the results of endobronchial ultrasound-guided transbronchial needle aspiration in making diagnosis of mediastinal injuries associated to different causes.

**Methods:**

A retrospective cross-sectional study of patients submitted to Endobronchial ultrasound-guided transbronchial needle aspiration at a private organization, between June 2013 and October 2016. All cases referred for collection of lymph nodes or peritracheal/peribronchial masses by endobronchial ultrasound-guided transbronchial needle aspiration, and evaluated through tomography or PET-CT were included. Interventional pulmonologists and thoracic surgeons with experience in the method did the procedures. Rapid on-site evaluation of fine needle aspiration was performed by an experienced pathologist. Material analysis included cytological smear and cytopathological analysis of paraffin-embedded cell blocks. Other specific analyses (immunocytochemistry, tests and cultures of infectious agents) were performed whenever necessary.

**Results:**

We included 72 patients; 6 were excluded for presenting endobronchial lesions in which bronchoscopic biopsy could be performed, or intrathoracic lesions that were not accessible by endobronchial ultrasound-guided transbronchial needle aspiration. The mean age of 66 patients included for analysis was 61.17 years (±14.67 years), with a predominance of males (64%). Endobronchial ultrasound-guided transbronchial needle aspiration was definitive for diagnosis in 60 cases (91%). Three cases (4.5%) had inconclusive test results. There were no major complications related to the procedure.

**Conclusion:**

Endobronchial ultrasound-guided transbronchial needle aspiration had a high diagnosis yield, with minimal morbidity, being an excellent option for diagnostic approach of patients with lymphadenopathy or intrathoracic lesions, and for neoplasm staging.

## RESUMO

**Objetivo:**

Descrever os resultados da utilização de punção aspirativa por agulha guiada por ultrassom endobrônquico na definição do diagnóstico em casos de lesões mediastinais por diferentes causas.

**Métodos:**

Estudo transversal retrospectivo de pacientes submetidos à punção aspirativa por agulha guiada por ultrassom endobrônquico em instituição privada, entre junho de 2013 e outubro de 2016. Foram incluídos todos os casos referenciados para coleta de materiais de linfonodos ou massas peritraqueais/peribrônquicas por punção aspirativa por agulha guiada por ultrassom endobrônquico, estudados com tomografia ou PET-CT. Os procedimentos foram realizados por pneumologistas intervencionistas e cirurgiões torácicos com experiência no método. *Rapid on-site evaluation* da punção aspirativa por agulha fina foi realizada por patologista experiente. A análise do material incluiu citologia dos esfregaços em lâminas e análise citopatológica do emblocado celular. Outras análises específicas (imunocitoquímica, pesquisas e culturas de agentes infecciosos) foram realizadas se necessárias.

**Resultados:**

Foram incluídos 72 pacientes; destes, 6 foram excluídos por apresentarem lesões endobrônquicas passíveis de biópsia broncoscópica convencional ou lesões intratorácicas não acessíveis a punção aspirativa por agulha guiada por ultrassom endobrônquico. Assim, a média de idade dos 66 pacientes incluídos para análise foi 61,17 anos (±14,67 anos), com predomínio do sexo masculino (64%). A punção aspirativa por agulha guiada por ultrassom endobrônquico foi definitivo para o diagnóstico em 60 casos (91%). Três casos (4,5%) foram inconclusivos. Não houve complicações maiores relacionadas ao procedimento.

**Conclusão:**

A punção aspirativa por agulha guiada por ultrassom endobrônquico teve elevado rendimento diagnóstico, com mínima morbidade, constituindo excelente opção na abordagem diagnóstica de pacientes com linfadenopatia ou lesões intratorácicas e no estadiamento de neoplasias.

## INTRODUCTION

Access to the mediastinum can pose significant technical challenges. The 1980's consolidated mediastinoscopy as a safe way of accessing the mediastinum and a gold standard for non-invasive approach to the paratracheal compartment. With a high accuracy (greater than 80%) and very low morbidity and mortality, the method is safe and available in most centers specializing in chest diseases.^(^ ^1^ ^)^ In the last two decades, however, the technological advancement of endoscopic and video-assisted techniques has broadened the range of options to address the mediastinum.^(^ ^2^ ^)^

Endobronchial ultrasound-guided transbronchial needle aspiration (EBUS-TBNA) should be considered among the minimally invasive techniques available for diagnosis of lymphadenopathies and/or mediastinal masses of different etiologies, as well as for staging of pulmonary and extrapulmonary neoplasms. Its main advantages include the possibility of getting access by real time puncturing, with direct observation of lesions and regional vessels, thus increasing the chances of appropriate collection and minimizing the risk of significant bleeding.

Endobronchial ultrasound-guided transbronchial needle aspiration is performed with a dedicated bronchoscope with an ultrasound transducer at the tip, enabling viewing of peribronchial or peritracheal lesions (lymph nodes or masses). The equipment allows the use of a dedicated aspiration needle for targeted collection of cytological material from these lesions.

The use of EBUS-TBNA has increased since the technique was described in the 1990's.^(^ ^3^ ^)^ Overall, studies have demonstrated its efficacy and safety in different situations, particularly in the management of patients with lung cancer, for whom surgical interventions, such as mediastinoscopy or video access or thoracotomies, could be avoided.^(^ ^4^ ^)^

In Brazil, the first study with EBUS-TBNA was performed in patients with esophageal cancer, in 2009,^(^ ^5^ ^)^ to assess involvement of the airway wall by the tumor. Subsequently, the clinical use of EBUS-TBNA was extended to patients with lung cancer and other mediastinal diseases.^(^ ^6^ ^)^ Despite the excellent results observed in the international literature, the cost and the small amount of equipment installed in different organizations limit the widespread use of the method, as well as the number of publications on the subject in Brazil.

## OBJECTIVE

To describe the results of using endobronchial ultrasound-guided transbronchial needle aspiration to determine the diagnosis in cases of mediastinal lesions of different causes and, based on these results, to offer subsidies for rational use of the method to approach the mediastinum.

## METHODS

A retrospective cross-sectional study based on the prospective database of the Respiratory Endoscopy Center of a large private hospital in the city of São Paulo, SP, Brazil. The study was previously approved by the Institutional Review Board of the organization, CEP: 11/1501.

We enrolled all cases referred for EBUS-TBNA between June 2013 and October 2016 with evidence of enlarged mediastinal lymph nodes or expansive intrathoracic lesions in peribronchial or peritracheal sites, aiming to diagnose these conditions and/or stage pulmonary or extrapulmonary neoplasms.

Before the examination, all patients underwent a clinical evaluation with a chest computed tomography (CT) scan and/or positron emission tomography/computed tomography (PET/CT) for planning of the procedure. Preparation included informing about the risks and benefits of the procedure and a signed informed consent. The procedures were performed by interventional pulmonologists and thoracic surgeons, who were skilled and experienced in the method.

### Endobronchial ultrasound-guided transbronchial needle aspiration and identification of lymph node chains

All procedures were performed under general anesthesia with laryngeal mask or tracheal intubation, and topical anesthesia of the tracheobronchial mucosa with 1% lidocaine. The equipment used was Olympus BFUC180F and EU-ME1 image processor (Olympus Medical Systems, Tokyo, Japan).

Standardization of the procedure included initial evaluation of the tracheobronchial tree by video-bronchoscopy to rule out potential endobronchial lesions whose presence could determine diagnosis or management, followed by EBUS-TBNA. Lymph node stations were analyzed according to the lymph node map recommended by the International Association for the Study of Lung Cancer (IASLC)^(^ ^7^ ^)^ in a standard way, covering the right and left superior paratracheal stations (2R, 2L), as well as the inferior (4R and 4L), subcarinal,^(^ ^7^ ^)^ right and left peribronchial (10R and 10L), and right and left hilar (11R, 11L).

For staging of pulmonary tumors, the protocol included systematic evaluation of lymph node chains N3, N2 and N1, sequentially (from the least probable to the most probable topography), based on the lung topography of the originating lesion, associated with analysis by CT scan and/or PET/CT. Lymph nodes suspected of involvement by neoplasms on ultrasound (rounded, heterogeneous, with areas of necrosis and defined margins), according to the classification of Fujiwara et al.,^(^ ^8^ ^)^ were aspirated and sent for cytopathological analysis, which included smears on slides and cell block, in addition to other complementary studies, such as immunocytochemistry, specific staining for investigation of infectious agents, and screening of tumor biomarkers, according to the clinical suspicion.

In cases requiring diagnosis of a mediastinal lesion or staging of extrathoracic neoplasms, lymph nodes or masses were chosen based on review of the chest CT scan and/or PET/CT. Lymph nodes over 1cm in their smallest diameter and/or those with anomalous fluorine-18 fluorodeoxyglucose (FDG) uptake and considered suspect upon examination were addressed by TBNA.

### Aspiration procedure, cytopathological analysis and complements

For staging of pulmonary neoplasms, aspiration was performed on PET/CT avid and non-avid lymph nodes, when they showed morphological characteristics suggestive of malignancy on ultrasound. This classification considers the morphology, heterogeneity, vascularization and margins of mediastinal lesions on ultrasound.^(^ ^8^ ^)^

After identification and analysis of lymph node basins using EBUS-TBNA, fine-needle aspiration (FNA) of suspected lesions was carried out in real time with the Vizishot 22 Gauge needle (Olympus Medical Systems). A minimum of three aspirations were performed on target lesions or lymph nodes.

**Figure 1:**
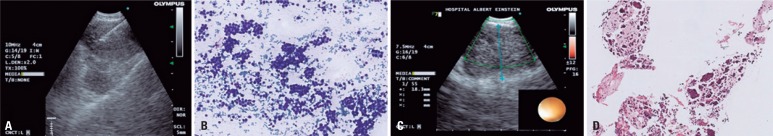
(A) Lymph node aspiration by endobronchial ultrasound-guided transbronchial needle aspiration. (B) Cytological smears show atypical epithelial cell clusters in a lymphoid background. (C) Heterogeneous lymph node seen on endobronchial ultrasound-guided transbronchial needle aspiration. (D) Cell block with hystiocytic aggregates forming granulomas

To assess the representativeness of the collected material, rapid on-site evaluation (ROSE) was systematically carried out on the material aspirated by a pathologist. After confirmation of adequate qualitative and quantitative cellularity (representative material), additional aspirates were fixed in formalin and sent to the pathology laboratory for subsequent cell block preparation and analysis (Figure 1). According to each specific case, additional materials were collected for other laboratory tests (flow cytometry/immunophenotyping, cultures and specific screening). According to the pathological anatomy report, needle track, hemorrhagic or low-cellularity materials were considered as non-representative and inadequate for definitive diagnosis.

In cases of suspected sarcoidosis, neoplastic infiltration or inflammatory/infectious disease, other samples were collected in addition to the EBUS-TBNA, namely: bronchoalveolar lavage (BAL) for cultures and/or differential cell count and CD4/CD8, and transbronchial or endobronchial biopsies aiming to improve the diagnostic performance of the procedure.

To assess the sensitivity of EBUS-TBNA, we considered as conclusive those cases of malignant neoplasms confirmed by cytopathology, with no neoplasms confirmed by surgery or long-term follow-up (at least 6 months), with inflammatory or infectious diseases defined on cytopathology and consistent with the clinical/radiological picture and, finally, cases with reactive lymph nodes consistent with the clinical/radiological picture, and with long-term follow-up.

## RESULTS

Initially, 72 patients were reviewed; 6 were excluded for having endobronchial lesions not requiring EBUS-TBNA for diagnosis or staging, or lesions that were inaccessible to the method. Thus, 66 patients were included in the final analysis. The mean age was 61.17±14.67 years, 64% of males. Patient characteristics are summarized in table 1.

**Table 1:** General characteristics of patients subjected to endobronchial ultrasound-guided transbronchial needle aspiration and investigated lesions

Characteristics		Results n (%)
Male		42 (64)
Age		61.2 (14.7)
Indications for the examination		
	Diagnosis or staging of pulmonary neoplasm	34 (52)
	Intrathoracic lymphadenopathy in other neoplasms	23 (35)
	Intrathoracic lymphadenopathy to clarify	9 (14)
Size lymph nodes or lesions, smaller diameter (mm)		15.2 (7.5)
Localization of enlarged lymph nodes/lesions		
2 lymph node stations		31 (47)
	Multiple lymph node stations	26 (39)
	Subcarinal, isolated	7 (11)
	Paratracheal, isolated	2 (3)

Results expressed in n (%) or mean (standard deviation).

Endobronchial ultrasound-guided transbronchial needle aspiration was indicated for diagnosis and/or staging of lung cancer in 52% of cases, suspected recurrence of extrathoracic neoplasms in 23% of cases, intrathoracic lymphadenopathy in 14% of cases, and intrathoracic lymphadenopathy in patients with multiple neoplasms and/or lymphomas in 12% of cases.

Of 57 patients with neoplasms for diagnosis or staging, or with lesions suspected of thoracic or extrathoracic neoplasms, malignant neoplasm was confirmed in 67%. Of those with mediastinal lymphadenopathy, the exam was positive for neoplasm in only one case.

### Analysis of aspirates

**Figure 2:**
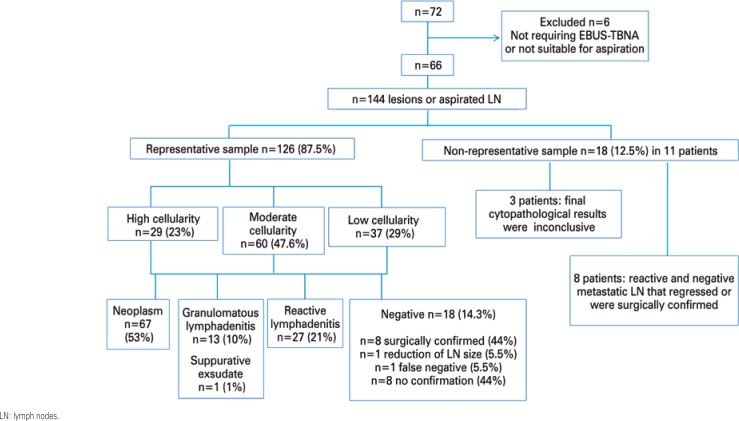
Representativeness of the material aspirated during endobronchial ultrasound-guided transbronchial needle aspiration

A total of 144 lymph nodes/lesions (mean of 2.16±1.4 per patient) were aspirated, averaging 15.15±7.5mm in their smallest diameter and 19.3±3.5mm in their largest diameter. Lymph node clusters were observed in 18 patients (27%), making it impossible to acquire objective measures. By the ROSE evaluation, 87.5% of the material obtained was representative of lymph nodes/lesions, with moderate and high cellularity in 71%, and low cellularity in 29%. In 12.5% (n=18 lymph nodes/lesions) of the cases, the material obtained was not representative, but rather needle track, hemorrhagic or low cellularity materials (Figure 2).

Of all lymph nodes/lesions aspirated, 53% were neoplasms (n=67), 10% granulomatous lymphadenitis (n=13), 21% reactive lymphadenitis (n=27), and 14.3% (n=18) were negative. Of 18 lymph nodes/lesions with negative results, in 44.4% (n=8) this was confirmed by surgery; in 5.5% (n=1) there was reduction in size and metabolic activity over the 6 months of radiological evolution, and in 5.5% (n=1) the result was a false-negative (patient was diagnosed with lymphoma in a subsequent analysis of surgical material). In 44.4% (n=8), no results were obtained by other methods. Of 13 lymph nodes with characteristics of granulomatous lymphadenitis, in 23% (n=3) a diagnosis of sarcoidosis was suggested; histoplasmosis was diagnosed in 7.7% (n=1), and the remaining 69% (n=9) persisted as nonspecific granulomatous lymphadenitis (Figure 2).

Of 27 reactive lymph nodes, 33% (n=9) were confirmed by surgery, 15% (n=4) were confirmed by biopsies in other lymph node sites, 30% (n=6) showed a decrease or no change in size within 6 months of follow-up, and 30% (n=8) were not confirmed by other methods.

### Analysis of individual patient results

The final EBUS-TBNA results based on the overall individual pathological examinations were pulmonary or extrapulmonary neoplasm in 59% of cases, granulomatous or inflammatory/infectious disease in 12%, confirmed reactive lymphadenopathy in 14%, and confirmed negative lymph nodes in 6%. These results add up to a diagnostic performance of 90.9%.

**Figure 3:**
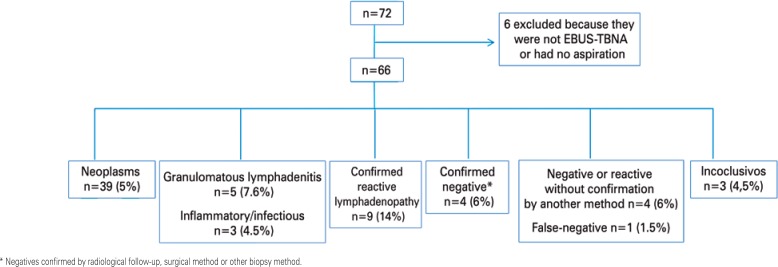
Diagnoses obtained by endobronchial ultrasound-guided transbronchial needle aspiration in study subjects

In 11 patients, we found 18 non-representative lymph nodes/lesions. Of these, three had inconclusive results (without a definitive cytopathological diagnosis); four had metastases diagnosed in other intra or extrathoracic lymph node sites; two had a diagnosis of reactive lymphadenitis (one was surgically confirmed and one was not followed up); and two had negative lymph nodes (one was surgically confirmed and one showed regression at follow-up) (Figure 3).

Most of the patients (80%) had the EBUS-TBNA performed in an outpatient setting, remaining in postanesthetic recovery for approximately one to two hours after the examination, and being discharged with recommendations for post-anesthesia care and bleeding events.

No major complications related to the EBUS-TBNA procedure were observed. There was one case of major oropharyngeal discomfort related to the laryngeal mask, which improved with analgesic agents. All patients were discharged without major clinical complications.

## DISCUSSION

Minimally invasive access to the mediastinum can be obtained through video-surgery, mediastinoscopy, CT-guided biopsy, or EBUS-TBNA. These methods may be considered complementary when, for instance, definitive surgical therapy is required. The complications and morbidity related to more invasive methods can be avoided when the biopsy is merely diagnostic, or in conclusive cases of EBUS-TBNA for the staging of neoplasms.

There are few studies published in the Brazilian literature on the diagnostic performance of bronchoscopy with EBUS-TBNA-guided biopsy.^(^ ^6^ ^,^ ^9^ ^)^ This study is the first analysis of EBUS-TBNA results performed exclusively in a private hospital in the State of São Paulo since the method was introduced in our country. The importance of analyzing the results of these procedures is to assess the quality and implement improvements to consolidate the success of the method.

Endoscopic ultrasound-guided fine needle aspiration with a sector transducer has been established as a method of choice for diagnosis of mediastinal pathologies in peribronchial or peritracheal topographies, and for staging of lung cancer and extrathoracic neoplasms.^(^ ^10^ ^,^ ^11^ ^)^ We emphasize the importance of a clinical-radiological evaluation before the examination, which allows for adequate planning of the procedure. It is also important to know the regional topographies to be approached and the TNM classification, particularly for pulmonary neoplasms. The systematization of the exam also leads to an appropriate and effective approach, as well as a uniform language, which facilitates interpretation of test results and proper management of the patient. Sequential access to the N3, N2, N1 chains avoids contamination with tumor cells from one mediastinal site to the other, ensuring quality and effectiveness of results.

It is known that EBUS-TBNA does not enable collection of samples from 100% of lymph node chains affected by non-small cell carcinoma. In this regard, a meta-analysis published in 2013 showed high sensitivity and specificity when EBUS-TBNA was combined with endoscopic ultrasound-guided fine needle aspiration (EUS-FNA), since they are complementary tests, particularly for sampling of chains 8 and 9 of the lymph node map described above.^(^ ^12^ ^)^ Exception is made to chain 5 (aortopulmonary window), for which video-surgery is more suitable.^(^ ^2^ ^)^

Evaluation of the material by pathologists is another important part of the procedure to attest the representativeness of the aspirated material. This contributes to reducing the number of additional procedures for initial diagnosis of intrathoracic lesions and collection of lymph node stations, for example, in staging of pulmonary neoplasms, since patients with compromised N3/N2 chains would generally not require additional collections in other chains and would be referred for specific cancer treatment.^(^ ^13^ ^)^

In our group of patients, most exams were for diagnosis or staging of intra- or extrathoracic neoplasms. Of these patients, most had confirmed neoplasms, followed by other diagnoses (granulomatosis, infection and inflammation), which shows the importance of differential diagnosis in these cases.

Our study showed a diagnostic yield of about 91%, considering only the cases confirmed on examination, and negative or reactive cases confirmed by other diagnostic methods or clinical-radiological follow-up. This is consistent with international studies showing sensitivity between 84 and 96%.^(^ ^12^ ^,^ ^14^ ^–^ ^18^ ^)^ Of the total number of negative and reactive lymph nodes, 50% of negative results were confirmed (by surgery or follow-up) and 70% of reactive inflammations were confirmed by surgery, biopsy of other sites and follow-up.

However, we believe that this study has limitations, since some patients were lost to follow up, making it impossible to collect postoperative or clinical data, particularly in those with negative or reactive lymph nodes. This was because our site is a diagnostic center and we receive patients from external physicians located in different places in the country. These factors certainly hinder a more accurate final analysis of negative lymph nodes.

Finally, when investigating the representativeness of the materials obtained by aspiration site, inconclusive results occurred in patients who had a few non-representative samples, and representative samples increase the chance of a conclusive diagnosis. It is important to emphasize that, the more lesions or lymph nodes we aspirate per patient, the greater the chance of a conclusive diagnosis, which minimizes the effects of inconclusive samples collected from certain sites.

Our study had approximately 5% of tests with inadequate results (inconclusive) and 5.5% of false negatives, consistent with the literature.^(^ ^9^ ^,^ ^19^ ^,^ ^20^ ^)^

Other limitations of the study were related to the retrospective analysis based on a database, subject to errors in the recording of exams, such as CT scan and specific clinical data. However, even with the abovementioned limitations, the results of the study point to the need for continuous improvement of test results.

## CONCLUSION

Endobronchial ultrasound-guided transbronchial needle aspiration showed high diagnostic yield and low complication rates, representing an effective procedure for diagnosing different types of intrathoracic lesions as well as for the staging of neoplasms.
