# *Erratum:* Endobronchial ultrasound-guided transbronchial needle aspiration (EBUS-TBNA) in diagnosis of mediastinal lesions

**DOI:** 10.1590/S1679-45082018AO4094E

**Published:** 2018-07-30

Ricardo Sales dos Santos^1^, Marcia Jacomelli^1,2^, Juliana Pereira Franceschini^3^, Iunis Suzuki^1^, Altair da Silva Costa Jr.^1,4^, Christina Shiang^1^, Addy Lidvina Mejia Palomino^1^

^1^ Hospital Israelita Albert Einstein, São Paulo, SP, Brazil.

^2^ Hospital das Clínicas, Faculdade de Medicina, Universidade de São Paulo, São Paulo, SP, Brazil.

^3^ Centro Universitário São Camilo, São Paulo, SP, Brazil.

^4^ Universidade Federal de São Paulo, São Paulo, SP, Brazil.

In the manuscript “Endobronchial ultrasound-guided transbronchial needle aspiration (EBUS-TBNA) in diagnosis of mediastinal lesions”, DOI number: 10.1590/S1679-45082018AO4094, published at einstein (São Paulo). 2018;16(2):1-7, page 5, figure 3:

**Stated:**

Neoplasms n=39 (5%)

**It should be read:**

Neoplasms n=39 (59%)

